# Effectiveness of a WHO Safe Childbirth Checklist Coaching-based intervention on the availability of Essential Birth Supplies in Uttar Pradesh, India

**DOI:** 10.1093/intqhc/mzy086

**Published:** 2018-04-30

**Authors:** Jenny J Maisonneuve, Katherine E A Semrau, Pinki Maji, Vinay Pratap Singh, Kate A Miller, Ian Solsky, Neeraj Dixit, Jigyasa Sharma, Janaka Lagoo, Natalie Panariello, Brandon J Neal, Tapan Kalita, Nabihah Kara, Vishwajeet Kumar, Lisa R Hirschhorn

**Affiliations:** 1 Ariadne Labs, Brigham and Women’s Hospital, Harvard T.H. Chan School of Public Health, Boston, MA, USA; 2 Division of Global Health Equity, Brigham and Women’s Hospital, Boston, MA, USA; 3 Department of Medicine, Harvard Medical School, Boston, MA, USA; 4 Population Services International, Lucknow, Uttar Pradesh, India; 5 Department of Global Health and Population, Harvard T.H. Chan School of Public Health, Boston, MA, USA; 6 Community Empowerment Lab, Lucknow, Uttar Pradesh, India; 7 Department of Medical Social Sciences, Feinberg School of Medicine, Northwestern University, Chicago, IL, USA

**Keywords:** supply availability, maternal and newborn health, WHO Safe Childbirth Checklist, quality improvement

## Abstract

**Objective:**

Evaluate the impact of a World Health Organization Safe Childbirth Checklist coaching-based intervention (BetterBirth Program) on availability and procurement of essential childbirth-related supplies.

**Design:**

Matched pair, cluster-randomized controlled trial.

**Setting:**

Uttar Pradesh, India.

**Participants:**

120 government-sector health facilities (60 interventions, 60 controls). Supply-availability surveys were conducted quarterly in all sites. Coaches collected supply procurement sources from intervention sites.

**Interventions:**

Coaching targeting implementation of Checklist with data feedback and action planning.

**Main Outcome Measures:**

Mean supply availability by study arm; change in procurement sources for intervention sites.

**Results:**

At baseline, 6 and 12 months, the intervention sites had a mean of 20.9 (95% confidence interval (CI): 20.2–21.5); 22.4 (95% CI: 21.8–22.9) and 22.1 (95% CI:21.4–22.8) items, respectively. Control sites had 20.8 (95% CI: 20.3–21.3); 20.9 (95% CI: 20.3–21.5) and 21.7 (95% CI: 20.8–22.6) items at the same time-points. There was a small but statistically significant higher availability in intervention sites at 6 months (difference-in-difference (DID) = 1.43, *P* < 0.001), which was not seen by 12 months (DID = 0.37, *P* = 0.53). Greater difference between intervention and control sites starting in the bottom quartile of supply availability was seen at 6 months (DID = 4.0, *P* = 0.0002), with no significant difference by 12 months (DID = 1.5, *P* = 0.154). No change was seen in procurement sources with ~5% procured by patients with some rates as high as 29% (oxytocin).

**Conclusions:**

Implementation of the BetterBirth Program, incorporating supply availability, resulted in modest improvements with catch-up by control facilities by 12 months. Supply-chain coaching may be most beneficial in sites starting with lower supply availability. Efforts are needed to reduce reliance on patient-funding for some critical medications.

**Trial Registration:**

ClinicalTrials.gov #NCT02148952; Universal Trial Number: U1111-1131–5647

## Introduction

Reducing childbirth-related maternal and neonatal morbidity and mortality requires improving patients’ access to high quality maternity care [1–3]. Although rates of facility-based deliveries have increased, this shift has not consistently translated into improved outcomes [4–7]. In 2012, the Neonatal Mortality Rate in Uttar Pradesh, India remained unacceptably high at 49 deaths per 1000 live births [8, 9].

In an effort to improve the quality and outcomes of maternity care and in collaboration with the Government of India and State of Uttar Pradesh, a randomized controlled trial was undertaken to measure the impact of the BetterBirth Program, a coaching-based implementation of the World Health Organization (WHO) Safe Childbirth Checklist [10–13]. The Checklist is a job aid designed to help birth attendants adhere to 28 essential birth practices known to save lives [14].

Adherence to the Checklist-identified practices requires 28 supplies (Table 1). However in India, there are significant gaps in the availability of medicines and equipment for maternity care in government-sector facilities [15–17]. For example, a survey in Uttar Pradesh found that only 53.8% of first-line referral facilities had injectable magnesium sulfate available [17]. When supplies are unavailable in facilities, patients either buy them or forego treatment. As a result, the unavailability of supplies is a serious barrier to the provision of high quality care in facilities and, consequentially, a positive outcome for mothers and babies of all socioeconomic backgrounds [18].

**Table 1:** Supplies associated with WHO Safe Childbirth Checklist essential birth practices, location of evaluated items, and whether their source of procurement was evaluated in the BetterBirth trial in Uttar Pradesh, India

Components of checklist supply score (28 items total)	Associated essential birth practice on the WHO Safe Childbirth Checklist	Location evaluated for availability^a^ (1- admission, 2- duty room, 3- labor and delivery (L&D) room, 4- post-partum room or anywhere in facility (Facility))	Evaluated for procurement (*N* = 23)^b^
*Pause Point 1*			
1. Thermometer	Mother’s temperature^c^	All four locations	X
2. Blood pressure cuff	Mother’s blood pressure^c^	All four locations	X
3. Stethoscope			X
4. Fetoscope or Doppler	Fetal heart rate^c^	Admission room, Duty room, L&D room	X
5. Partograph	Partograph started	Admission room, Duty room, L&D room	
6. Antibiotics (mother)	Antibiotics for the mother^c^	Facility	X
7. Urine dip sticks	Magnesium sulfate for the mother^c^	Facility	X
8. Magnesium sulfate		All four locations	X
9. HIV testing kit	HIV status of the mother	Facility	
10. Nevirapine (mother)	Administer nevirapine as needed	Facility	
*Pause Point 2*			
11. Clean gloves	Confirm essential supplies for mother are at bedside	Admission room, Duty room, L&D room	X
12. [(Soap and		Admission room, Duty room, L&D room	X
12. Clean water) or		Admission room, Duty room, L&D room	X
12. (Alcohol rub)]		Admission room, Duty room, L&D room	
13. Sterile needle/syringe		All four locations	X
14. Oxytocin		L&D room	X
15. Clean pads		Admission room, Duty room, L&D room	X
16. Clean towel	Confirm essential supplies for baby are at beside^c^	L&D room	X
17. Clean blade/scissor		L&D room	X
18. Cord tie/clamp		L&D room	X
19. Mucus extractor/suction		L&D room	X
20. Bag-and-mask		L&D room	X
21. Vitamin K		Facility	X
*Pause Point 3*			
1. Thermometer	Baby’s temperature^c^	All four locations	Above
3. Stethoscope	Baby’s respiratory rate^c^	All four locations	Above
22. Baby scale	Baby’s weight	L&D room	X
23. Intravenous fluid bag	Start IV fluids (if mother bleeding)	Facility	
24. Antibiotics (baby)	Antibiotics for the baby^c^	Facility	X
25. Baby warmer	Special care/monitoring for baby	L&D room	
26. Nevirapine (baby)	Administer nevirapine as needed	Facility	
*Pause Point 4*			
27. BCG vaccine	Administer vaccines	Facility	X
28. Polio vaccine		Facility	X

BCG, *Bacillus* Calmette–Guérin Vaccine; L&D, Labor and Delivery.

^a^Item is deemed available if it is present in a designated area and functional (or not expired, as relevant). While certain items (e.g. stethoscope or thermometer) can be available within any room in the labor ward complex (e.g. availability in either admission or duty room, or labor and delivery room, or post-partum ward), other items were expected to be available in a designated area (e.g. neonatal bag-and-mask available in the labor and delivery room).

^b^Procurement is how essential medicines and medical supplies are sourced. The source of supply was defined by four categories; Official: (1) district or state government defined system; (2) from facility funds; Unofficial: (3) Patient or family: brought from home or purchased or (4) Any other means, e.g. staff purchased or donation.

^c^Denotes a checklist practice repeated across multiple Pause Points. For simplicity, these are listed in this table only at the first Pause Point at which they are performed.

To avoid creating a non-sustainable supply source in the context of the study, the BetterBirth Program did not provide any supplies to facilities [13]. Instead, the theory of change was that coaching at the point of care, as well as at facility and district levels would improve supply availability by promoting behavior and system change through (a) improved knowledge of the essential supplies for childbirth and advocacy at the frontline to improve availability; (b) routine monitoring and data driven communication of supply gaps to facility and district leaders; accompanied by (c) coaching on action plans for solutions which leveraged existing supply chains and facility and district resources.

Although there have been efforts to identify interventions that improve health systems’ supply-chains, further research is needed to understand how supply availability through strengthening existing systems can be effectively integrated into coaching-based quality improvement efforts [15, 19, 20]. Here, we describe the BetterBirth Program’s impact on essential birth supply availability and the procurement source of available supplies.

## Methods

### Study design and site characteristics

The BetterBirth Trial was a matched pair, cluster-randomized controlled trial across 120 government health facilities in Uttar Pradesh, India conducted from October 2014 to January 2017 (Appendix A). The trial included primary, community and first-referral health centers (60 intervention and 60 control facilities). Detailed descriptions of the BetterBirth Program and trial methodology are described elsewhere [13, 21, 22].

### BetterBirth Program

Core to the BetterBirth intervention was a coaching team who worked to address non-adherence to essential birth practices, including supply-related barriers, at multiple healthcare system levels. At each of the 60 intervention facilities, coaches (nurses) conducted 43 day-long visits over 8 months (twice per week tapering to monthly) during which they worked with birth attendants to deliver Checklist-identified practices. Coaches provided feedback to birth attendants about their adherence, engaged them in identifying barriers and assisted with action planning to resolve issues. Coach Team Leaders (physicians or public health professionals) accompanied coaches on alternating visits, working with facility leadership to review data on Checklist adherence. They then worked with leadership to identify and resolve facility-level barriers to Checklist adoption, including improving supply availability. They also coached the Childbirth Quality Coordinator, a facility-based champion responsible for motivating staff utilization of the Checklist, identifying and resolving supply issues and supporting the facility’s commitment to Checklist-use post-trial.

In addition to one-on-one coaching, Coaches and Coach Team Leaders held data-sharing meetings at facilities fortnightly to feedback data on Checklist adherence and supply availability and support action planning. To ensure district-level support, Coach Team Leaders facilitated bi-monthly progress meetings with district health officials and facility leadership to review adherence and supply-related data and help identify plans needed at the district level to improve childbirth care, including supply availability. The BetterBirth Program did not provide supplies (except paper copies and posters of the Checklist during the trial) or financial support. Results of the impact BetterBirth had on essential birth practice uptake and health outcomes are described elsewhere [22, 23].

### Ethics

The study protocol received approval by the following ethical review boards at the Community Empowerment Lab, Jawaharlal Nehru Medical College, Harvard T.H. Chan School of Public Health, Population Service International, the WHO and the Indian Council of Medical Research. Each facility and birth attendant formally agreed to participate in the BetterBirth Program at the beginning of the study. Coaches accompanied birth attendants during their work-shift and documented practices during patient-care activities at the facility. Coaches collected no patient identifiers.

### Data sources

#### Facility essential birth supply availability survey

Study staff conducted the supply-availability survey (Appendix D) at all sites to measure availability of the 28 essential birth supplies (Table 1). During unannounced visits, data collectors observed facility areas where supplies were supposed to be stocked. If an item was observed as functional, not expired, and in at least one of the designated areas, it was coded as available. Coaches were not present on days when surveys were conducted and did not have access to data. Data collectors conducted facility surveys at baseline and quarterly over the study. Baseline for intervention sites was just prior to the beginning of the intervention. Baseline for control sites was just prior to the first study-related visits, which occurred ~2 months after intervention start at their matched pair [22]. Surveys were collected from the 60 pairs at baseline through 6 months and from 58 pairs at 9 and 12 months (two facilities closed and moved after completing their third survey at 6 months, resulting in exclusion of these sites and their pairs from the 12 month analysis). For simplicity, only data from baseline, 6 and 12 months are reported; 3- and 9-month data are available upon request.

#### Coach supply source survey

During facility visits, Coach Team Leaders conducted a supply source survey (Appendix E) with the facility leader or Childbirth Quality Coordinator to measure the availability and procurement source of 23 of the 28 checklist supplies (Table 1). If an item was available, Coach Team Leaders would discuss with facility representatives how the supply was procured: (1) through the district or state government supply-system, (2) from facility funds, (3) from patient or family (brought from home or purchased) or (4) from any other means (e.g. facility staff purchased or donation). The first survey was conducted on the day after the BetterBirth Program launched and was repeated approximately every 2 weeks and then monthly, as Coach Team Leader visits decreased in frequency. After their visit, Coach Team Leaders entered data into a mobile phone-based CommCare application (Dimagi, Cambridge, MA), which visualized data over time as a heat map (Appendix F) to be shared with facility staff and leadership to inform action planning [13].

### Analysis of surveys

All statistical analyses were conducted using SAS v9.4 (SAS Institute, Cary, NC).

#### Facility essential birth supply-availability survey

The primary outcome was the difference in overall availability of essential birth supplies after 6 months of coaching. Six-months was chosen because maximum impact was expected at this time while coaching was still underway and a decay was seen in adherence to Checklist practices in intervention sites at 12 months (4 months after the intervention’s end) [22].

Essential birth supply availability was calculated as the count of the 28 Essential Birth Supplies available during a survey administration (Table 1). The mean percentage of sites with each supply available over time was calculated, in addition to the mean percentage of sites with all four medications considered critical to reducing maternal and neonatal harm (oxytocin, magnesium sulfate, Vitamin K and antibiotics for mothers or babies). To avoid inflating the analysis-wide α (type I) error, we did not conduct significance tests on these differences.

Within each matched pair, facility surveys could occur up to 2 months apart due to differences in baseline. Thus, models were constructed to estimate differences in supply levels across study arms at baseline, 6 and 12 months. Logistic regression models were fit using the counting method to predict the number of 28 items available, adjusting for matching and repeated measures over time [24]. Models included nonlinear terms for time and an interaction between study arm and time. From these models, the difference-in-difference (DID) was calculated for the number of supplies available from baseline to 6 months and, as a secondary outcome, from baseline to 12 months, with tests for statistical significance at *α* = 0.05.

To explore facilities starting with fewer essential birth supplies, a sub-analysis was conducted of facilities which fell into the bottom quartile of baseline supply availability (<20 of 28 supplies at baseline): 14 intervention sites (23%) and 14 control sites (23%). An additional model was fit that included a control for baseline supply availability interacted with study arm. The term would be significant if the intervention had a different effect among sites with fewer baseline supplies as compared to those with more.

#### Coach supply source survey

The proportion of available supplies purchased by an unofficial procurement method (defined as being purchased by the patient/family or ‘other source’) versus the official supply chain (procured by facility or district) over time was examined. In cases where a supply was procured from both an unofficial and an official method, it was coded as procured through the unofficial method. For this analysis, coach supply source data was grouped into 1-month time periods (except for the final months 8 and 9 which were grouped together) and the mean proportion of all available items procured by each source was calculated.

## Results

### Facility essential birth supply availability

A total of 596 completed facility supply-availability surveys were available for analysis. At baseline, the intervention sites had a mean of 20.9 items (95% CI: 20.2–21.5), which improved to 22.4 (95% CI: 21.8–22.9) at 6 months and 22.1 (95% CI: 21.4–22.8) at 12 months (Table 2). The control sites started with a mean of 20.8 items (95% CI: 20.3–21.3), which increased to 20.9 (95% CI: 20.3–21.5) at 6 months and 21.7 (95% CI: 20.8–22.6) at 12 months. There was a small but statistically significant better improvement in the intervention versus control arm at 6 months (DID = 1.43 supplies, *P* < 0.001), which was no longer significant by 12 months (DID = 0.37 supplies, *P* = 0.53).

**Table 2:** Mean number of the 28 essential birth supplies (with 95% CI) by arm over time in (1) all sites and (2) in facilities starting in the bottom quartile of supply availability (<20 supplies at baseline)

	0 Months (baseline)		6 Months		12 Months		Difference-in-difference	
	Intervention (*N* = 60)	Control (*N* = 60)	Intervention (*N* = 60)	Control (*N* = 60)	Intervention (*N* = 58)	Control (*N* = 58)	0–6 Months	0–12 Months
(1) All sites	20.9 (20.2–21.5)	20.8 (20.3–21.3)	22.4 (21.8–22.9)	20.9 (20.3–21.5)	22.1 (21.4–22.8)	21.7 (20.8–22.6)	1.43; *P* < 0.001	0.37; *P* = 0.53
	0 Months (baseline)		6 Months		12 Months		Difference-in-difference	
	Intervention (*N* = 14)	Control (*N* = 14)	Intervention (*N* = 14)	Control (*N* = 14)	Intervention (*N* = 14)	Control (*N* = 14)	0–6 Months	0–12 Months
(2) Bottom quartile sites	17.6 (16.5–18.6)	18.3 (17.4–19.2)	22.0 (21.0–23.1)	18.7 (17.6–19.9)	21.8 (20.6–23.1)	21.0 (19.6–22.5)	4.00 *P* = 0.0002	1.51 *P* = 0.154

Note: At baseline and 6 months, all 60 intervention sites are included. At month 12, only 58 sites are included because two matched pairs were excluded from the study (due to closure) and month 12 surveys were not conducted.

**Figure 1:**
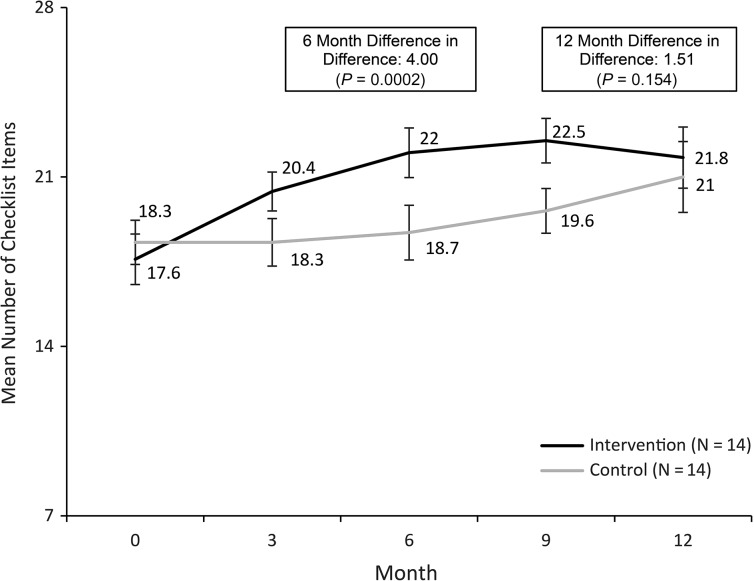
Mean number of Essential Birth Supplies (*N* = 28) over time by study arm for sites starting at the bottom quartile of supply availability (<20 of the 28 supplies at baseline).

The facilities with supply availability in the lowest quartile (<20 supplies) at baseline were spread across all five study-defined regions. While the average supply-availability score for intervention and control sites in this subgroup increased over time, intervention sites had significantly better improvement by 6 months (intervention: 17.6–22.0 for intervention and 18.3–18.7 for controls at baseline and 6 months, respectively, DID: 4.00, *P* = 0.0002) (Fig. 1 and Table 2). The difference in improvement between intervention and control was no longer statistically significant by 12 months (DID: 1.51, *P* = 0.154). Modeling results found that the interaction term between baseline supply level and study arm was statistically significant (*P* < 0.001, data not shown), confirming that the impact of the intervention was stronger among sites with lower baseline supplies.

Individual item supply availability was variable across sites. In the analysis of all sites, 18 items were highly available (≥80% of facilities) regardless of study arm at baseline and remained above that threshold throughout the study; five items had mid-range availability (50–79% of sites) throughout the study; and five items (partograph, nevirapine for baby, nevirapine for mother, Doppler or fetoscope and Vitamin K) had low availability (presence in <50% of facilities) at baseline (Table 3). While availability of Vitamin K and fetoscope/Doppler increased to mid-range in intervention but not control sites at month 6, the availability of partograph and nevirapine for mother and baby remained low in both arms (Table 3). For the bottom quartile intervention sites, eight items moved from mid-level availability at baseline to high availability at 6 months (Table 4). In contrast to the overall intervention sites, in the bottom quartile intervention sites, no increase from the low availability category was seen for the fetoscope/Doppler.

**Table 3:** Average availability of each of the 28 Essential Birth Supplies on the WHO Safe Childbirth Checklist across intervention and control sites over time

Average percentage of sites with each item available over time^c^							
Item	0 Months (baseline)		6 Months		12 Months		Category based on change in % of Intervention sites with item available from baseline to 6 months
	Intervention	Control	Intervention	Control	Intervention	Control	
	*n* (%)	*n* (%)	*n* (%)	*n* (%)	*n* (%)	*n* (%)	
	(*N* = 60)	(*N* = 60)	(*N* = 60)	(*N* = 60)	(*N* = 58)	(*N* = 58)	
Suction machine/mucus extractor	60 (100%)	60 (100%)	60 (100%)	59 (98.3%)	58 (100%)	57 (98.3%)	High baseline availability across intervention facilities (≥80%) that remains high (≥80%) at 6 months
Neonatal bag-and-mask	52 (86.7%)	55 (91.7%)	59 (98.3%)	56 (93.3%)	57 (98.3%)	54 (93.1%)	
Sterile blade	53 (88.3%)	48 (80.0%)	55 (91.7%)	56 (93.3%)	54 (93.1%)	51 (87.9%)	
Cord tie or clamp	54 (90.0%)	55 (91.7%)	55 (91.7%)	53 (88.3%)	52 (89.7%)	53 (91.4%)	
Hand hygiene supplies^a^	54 (90.0%)	52 (86.7%)	55 (91.7%)	53 (88.3%)	53 (91.4%)	51 (87.9%)	
Gloves	58 (96.7%)	59 (98.3%)	58 (96.7%)	58 (96.7%)	58 (100%)	57 (98.3%)	
IV Fluid	56 (93.3%)	57 (95.0%)	57 (95.0%)	56 (93.3%)	57 (98.3%)	57 (98.3%)	
Baby scale	59 (98.3%)	57 (95.0%)	60 (100%)	60 (100%)	58 (100%)	58 (100%)	
Pads	58 (96.7%)	55 (91.7%)	57 (95.0%)	57 (95.0%)	53 (91.4%)	56 (96.6%)	
Blood pressure instrument	50 (83.3%)	51 (85.0%)	59 (98.3%)	49 (81.7%)	51 (87.9%)	52 (89.7%)	
Stethoscope	56 (93.3%)	54 (90.0%)	60 (100%)	54 (90.0%)	57 (98.3%)	55 (94.8%)	
Urine dip sticks	54 (90.0%)	54 (90.0%)	54 (90.0%)	56 (93.3%)	53 (91.4%)	50 (86.2%)	
Sterile needle-syringe	58 (96.7%)	60 (100%)	59 (98.3%)	56 (93.3%)	57 (98.3%)	56 (96.6%)	
Antibiotics mother	53 (88.3%)	59 (98.3%)	57 (95.0%)	56 (93.3%)	56 (96.6%)	54 (93.1%)	
BCG vaccine	60 (100%)	59 (98.3%)	59 (98.3%)	60 (100%)	57 (98.3%)	58 (100%)	
Polio vaccine	60 (100%)	60 (100%)	58 (96.7%)	58 (96.7%)	57 (98.3%)	58 (100%)	
Baby warmer	58 (96.7%)	53 (88.3%)	56 (93.3%)	52 (86.7%)	50 (86.2%)	51 (87.9%)	
Thermometer	48 (80.0%)	51 (85.0%)	60 (100%)	46 (76.7%)	55 (94.8%)	51 (87.9%)	
Antibiotics baby	39 (65.0%)	36 (60.0%)	52 (86.7%)	42 (70.0%)	45 (77.6%)	47 (81.0%)	Mid-range availability that remains mid-range
Oxytocin	43 (71.7%)	47 (78.3%)	36 (60.0%)	45 (75.0%)	41 (70.7%)	35 (60.3%)	
Magnesium Sulfate	37 (61.7%)	30 (50.0%)	40 (66.7%)	34 (56.7%)	39 (67.2%)	32 (55.2%)	
HIV testing kit	35 (58.3%)	43 (71.7%)	42 (70.0%)	40 (66.7%)	41 (70.7%)	37 (63.8%)	
Clean towel	33 (55.0%)	28 (46.7%)	38 (63.3%)	29 (48.3%)	37 (63.8%)	33 (56.9%)	
Partograph	10 (16.7%)	7 (11.7%)	22 (36.7%)	15 (25.0%)	18 (31.0%)	14 (24.1%)	Low availability (<50%) that remains low
Nevirapine baby	2 (3.3%)	1 (1.7%)	3 (5.0%)	2 (3.3%)	2 (3.4%)	0 (0%)	
Nevirapine mother	4 (6.7%)	1 (1.7%)	3 (5.0%)	1 (1.7%)	3 (5.2%)	1 (1.7%)	
Fetoscope or doppler	23 (38.3%)	23 (38.3%)	41 (68.3%)	23 (38.3%)	47 (81.0%)	31 (53.4%)	Low availability (<50%) at baseline that moves to mid-range availability at 6 months
Vitamin K	16 (26.7%)	16 (26.7%)	32 (53.3%)	26 (43.3%)	37 (63.8%)	28 (48.3%)	
Percentage of sites with four critical medicines available^b^	9 (15.0%)	10 (16.7%)	18 (30.0%)	12 (20.0%)	24 (41.4%)	13 (22.4%)	

Note: At baseline and 6 months, all 60 intervention sites are included. However, at month 12, only 58 sites are included because two matched pairs were excluded from the study (due to closure) and month 12 surveys were not conducted.

^a^Presence of Hand hygiene supplies defined as either water and soap OR alcohol rub.

^b^Critical medications: Vitamin K, Magnesium Sulfate, Oxytocin, Antibiotics.

^c^For example, on average at baseline 26.7% of intervention sites and 26.7% control sites had Vitamin K available. At 6 months, 53.3% of intervention sites and 43.3% of control sites had Vitamin K available.

**Table 4:** Categorization of individual items based on change in percentage of sites with item available from baseline to 6 months for all intervention sites (*N* = 60) and the intervention sites starting with the lowest quartile of supply availability (<20 of 28 supplies, *N* = 14)

	High availability (≥80%) that remains high		Mid-range availability (50–79%) to high availability		Mid-range availability that remains mid-range		Low availability (<50%) to mid-range availability		Low availability that remains low	
	All intervention sites	Bottom quartile intervention sites	All intervention sites	Bottom quartile intervention sites	All intervention sites	Bottom quartile intervention sites	All intervention sites	Bottom quartile intervention sites	All intervention sites	Bottom quartile intervention sites
Suction machine	X	X								
Gloves	X	X								
Baby scale	X	X								
Pads	X	X								
Urine dip sticks	X	X								
Sterile needle-syringe	X	X								
BCG vaccine	X	X								
Polio vaccine	X	X								
Baby warmer	X	X								
Stethoscope	X			X						
Neonatal bag-and-mask	X			X						
Sterile blade	X			X						
Cord tie or clamp	X			X						
Hand hygiene supplies	X			X						
IV Fluid	X			X						
Blood pressure instrument	X			X						
Antibiotics mother	X			X						
Thermometer	X			X						
Antibiotics baby					X			X		
Oxytocin					X			X		
Magnesium Sulfate					X			X		
HIV testing kit					X			X		
Clean towel					X			X		
Vitamin K							X	X		
Fetoscope or doppler							X			X
Partograph									X	X
Nevirapine baby									X	X
Nevirapine mother									X	X

See Appendix B for actual percentages for each item. Note: At baseline and 6 months, all 60 intervention sites are included. However, at month 12, only 58 sites are included because two matched pairs were excluded from the study (due to closure) and month 12 surveys were not conducted.

Less than one-fifth of all sites had all four of the critical medications (oxytocin, magnesium sulfate, Vitamin K, antibiotics) available at baseline (15% and 16.7% in intervention and control arms, respectively) with availability improving at 6 months (30.0% and 20.0% in intervention and control arms, respectively) and 12 months (41.4% and 22.4% in intervention and control arms, respectively; Table 3).

#### Coach supply source survey

Over the course of the study, Coach Team Leaders completed 964 supply source surveys across the 60 intervention sites. Each facility was expected to have a minimum of 14 surveys completed; Coach Team Leaders completed more at their discretion (mean 16, range 9–23). As expected due to Coach Team Leader visits decreasing in frequency over the intervention, the total number of surveys completed per month declined from the first month of the intervention (*N* = 152) to the final months (*N* = 56). In the first month of the intervention, facilities procured 95% of available supplies from official sources (94% district, 1% facility) and 5% from unofficial sources (4% from patients and 1% from ‘other’ sources). No significant change was seen in the percentage of supplies sourced from official or unofficial sources over time (Appendix C). The top four supplies available due to purchase by patients were injectable oxytocin (29.1% purchased by patients), a sterile blade (28.4%), soap (23.1%) and Vitamin K (15.4%).

## Discussion

The BetterBirth Program integrated a focus on supply readiness and use into coaching to leverage existing supply chains and improve availability at the facility and point of care. On average, facilities started with relatively high supply levels. The intervention resulted in a modest but statistically significant higher mean overall availability of the required 28 supplies at 6 months. This difference was largely driven by increased availability of 2 supplies: fetal heart rate monitors and vitamin K, which increased from availability in <50% of facilities at baseline to between 50–79% of facilities at 6 months. However, this difference was no longer seen at 12 months, in part due to no further improvement in intervention sites along with increases in supplies in the control facilities including vitamin K, fetoscope/doppler and antibiotics for baby.

In this setting, coaching with data feedback and action planning at both district and facility levels was not enough to achieve a major and sustained impact on supply availability compared with temporal trends. Coaching was intended to address barriers in supply availability related to leadership engagement, birth attendant motivation and data feedback linked with action planning (Semrau and Maisonneuve, personal communication). However, there were likely other barriers less amenable to coaching that prevented a larger impact, such as those related to finances, policies, and district, state and national supply chain performance [15].

The WHO Safe Childbirth Checklist has been implemented in other global settings and the availability of essential supplies has remained a consistent barrier to performing the Essential Birth Practices [25]. Some coaching-based implementation models resolved supply barriers by providing supplies to facilities [26, 27]. Another WHO Checklist implementation model in Namibia did not provide supplies and found that availability improved over time with the coaching, however, availability of some items declined during the lower intensity ‘maintenance phase’ [28]. As noted, the BetterBirth Program did not provide supplies to facilities as we did not want to create an unsustainable supply-chain system in parallel to the state system.

Despite the intervention’s modest impact, the BetterBirth Program did result in a greater difference in availability among sites which started with lower baseline supply availability. This suggests that coaching may play a larger role in improving supply availability in facilities with weaker supply chain management. Facilities with higher baseline supply availability may not have much room for improvement in those supplies and supply chain gaps responsive to coaching and local change.

Further investigations are needed to understand why some supplies were responsive to coaching and why others were not. The intervention team noted in our discussions with them that supply-availability improvements were often seen when specific barriers amenable to district and facility coaching were resolved (for example, lack of knowledge of the importance of a supply or communication gaps in identifying and addressing shortages). We also found that for a single supply, there could be multiple site specific or broader barriers to availability. This may explain the heterogeneity seen in how the intervention improved availability of an item in some but not all sites (for example, Vitamin K). However, despite 6 months of coaching, less than one-third of intervention sites had all four critical medicines. This persistent gap highlights the need for broader supply chain remedies beyond coaching. We also found that supplies which were either infrequently needed (nevirapine in a low HIV seroprevalence setting) or remained at low uptake in intervention sites (partograph) did not respond to the intervention.

We did see some improvement in supply availability in control sites. It is possible that, while data collection visits were unannounced, the quarterly assessment of supplies improved awareness of the importance of supply status in control sites. Benefits to control sites may also have spread from the intervention due to district-level advocacy meetings with the Chief Medical Officer as some districts contained both control and intervention sites. There were also targeted state-wide programs focusing on health-facility assessments during the study that may have contributed to improvements such as Kaya Kalp, a National Rural Health Mission initiative focused on infection control [29]. These programs were incorporated at intervention and control sites.

In the intervention sites, while facilities procured an average of 95% of items assessed from official sources, patients provided nearly one-quarter of some important supplies including oxytocin, sterile blades and soap. This is consistent with other research demonstrating that although treatment at public facilities in India is supposed to be free, this is not always the case [30]. Further research on supply availability should capture procurement methods in greater detail to allow for better designed interventions that can improve the supply chain rather than relying on patients.

While other studies have also noted variation in supply availability across Uttar Pradesh and India, the global literature is less clear on interventions to address supply gaps that do not require implementing new supply chains or direct provision of resources. A systematic review analyzing the impact of interventions on medicine availability at the primary healthcare level found a variable degree of evidence and rigor [31]. Evidence was found that supervision visits in Zimbabwe strengthened primary health centers’ stock management though had limited effect on availability [32]. Comparatively, interventions focused on staff training programs on logistics management systems in Nepal showed improvements in supply availability and stockouts [33]. In our search, we did not find further evidence on the effectiveness of coaching interventions targeting supplies. Our findings suggest while coaching was able to catalyze improvements through changing some behaviors and actions in the existing system, additional supply chain strengthening interventions beyond knowledge and behavior change are needed to effectively address gaps in essential birth supplies and sustain improvement [15, 16].

This study had several limitations. While both surveys utilized drew from existing surveys and were pilot tested, they were not independently validated. However, we had formal data quality assurance protocols and trainings to support data collectors for the facility survey to ensure the quality of the data [34]. Additionally, we do not have information on the quantity of stock available or if it was appropriately stored. Thus, we may have overestimated the supply availability. Sources of procurement were also only by facility report and were not confirmed. Finally, we did not measure change in attitudes and culture related to supply availability so we cannot completely explain the successes and challenges encountered. Despite these limitations, our study is an important first step towards identifying opportunities and challenges for coaching to help impact supply availability.

## Conclusion

Integrating a focus on supply availability into coaching resulted initially in a modest increase in overall supply availability, with lower performing sites experiencing greater difference in availability after 6 months of the intervention. While coaching can play a role in strengthening supply availability, further research is needed to understand how it can be combined with broader supply-chain interventions to contribute to and sustain improvement in essential birth supply availability in frontline facilities in resource-limited settings.

## Supplementary Material

Supplementary Data
