# Supplementary material for: Effectiveness of a WHO Safe Childbirth Checklist Coaching-based intervention on the availability of Essential Birth Supplies in Uttar Pradesh, India
Source: Int J Qual Health Care. 2018 Apr 30;30(10):769–77. doi: 10.1093/intqhc/mzy086 (PMC6340347; doi:10.1093/intqhc/mzy086)
Supplement: Supplementary Data [file mzy086_supplies_appendices_resubmissionclean.docx]

**APPENDIX 1:** Baseline matching characteristics of health facilities included in the BetterBirth Trial

|  | **Intervention** | **Control** |
| --- | --- | --- |
| Number of facilities at baseline | 60 | 60 |
|  |  |  |
| Facility type (n, %) |  |  |
| Primary Health Center | 23 (38.3%) | 23 (38.3%) |
| Community Health Center | 27 (45%) | 29 (48.3%) |
| First Referral Unit | 10 (16.7%) | 8 (13.3%) |
|  |  |  |
| Distance to district hospital in km (mean, 95% CI) | 29.5 (25.9-33.1) | 30.3 (27.2-33.4) |
|  |  |  |
| Skilled birth attendants per facility (mean, 95% CI) | 4.4 (4.1-4.7) | 4.4 (4.1-4.7) |

| **APPENDIX 2 : Average availability of each Essential Birth Supply on the WHO Safe Childbirth Checklist across bottom quartile intervention and control sites over time (<20 baseline supplies)** | | | | | | | |
| --- | --- | --- | --- | --- | --- | --- | --- |
|  | | | | | | | |
| **Average percentage of sites with each item available over time**** | | | | | | | |
|  | **0 months (baseline)** | | **6 Months** | | **12 Months** | | **Categories based on change in percentage of intervention sites with item available from baseline to 6 months** |
| Item | **Intervention**  **(N=14)** | **Control**  **(N=14)** | **Intervention**  **(N=14)** | **Control**  **(N=14)** | **Intervention**  **(N=14)** | **Control**  **(N=14)** |  |
| Suction machine or mucus extractor | 14 (100%) | 14 (100%) | 14 (100%) | 13 (92.9%) | 14 (100%) | 13 (92.9%) | High availability across facilities (≥80%) that remains high |
| Pads | 14 (100%) | 14 (100%) | 14 (100%) | 13 (92.9%) | 14 (100%) | 14 (100%) |  |
| Gloves | 12 (85.7%) | 14 (100%) | 13 (92.9%) | 13 (92.9%) | 14 (100%) | 14 (100%) |  |
| Urine dip sticks | 13 (92.9%) | 12 (85.7%) | 13 (92.9%) | 13 (92.9%) | 13 (92.9%) | 10 (71.4%) |  |
| Sterile needle – syringe | 14 (100%) | 14 (100%) | 14 (100%) | 12 (85.7%) | 14 (100%) | 12 (85.7%) |  |
| BCG vaccine | 14 (100%) | 13 (92.9%) | 14 (100%) | 14 (100%) | 14 (100%) | 14 (100%) |  |
| Polio vaccine | 14 (100%) | 14 (100%) | 13 (92.9%) | 13 (92.9%) | 14 (100%) | 14 (100%) |  |
| Baby scale | 14 (100%) | 11 (78.6%) | 14 (100%) | 14 (100%) | 14 (100%) | 14 (100%) |  |
| Baby warmer | 12 (85.7%) | 11 (78.6%) | 13 (92.9%) | 11 (78.6%) | 12 (85.7%) | 11 (78.6%) |  |
| Stethoscope | 11 (78.6%) | 11 (78.6%) | 14 (100%) | 10 (71.4%) | 14 (100%) | 12 (85.7%) | Starts at mid-range availability (50-79%) across facilities moves to high availability (≥80%) at 6 months |
| Neonatal bag & mask | 11 (78.6%) | 13 (92.9%) | 14 (100%) | 12 (85.7%) | 14 (100%) | 13 (92.9%) |  |
| Sterile blade | 9 (64.3%) | 10 (71.4%) | 13 (92.9%) | 12 (85.7%) | 14 (100%) | 10 (71.4%) |  |
| Cord tie – clamp | 10 (71.4%) | 10 (71.4%) | 13 (92.9%) | 11 (78.6%) | 13 (92.9%) | 13 (92.9%) |  |
| Hand hygiene supplies^ | 11 (78.6%) | 9 (64.3%) | 13 (92.9%) | 10 (71.4%) | 13 (92.9%) | 12 (85.7%) |  |
| IV Fluid | 10 (71.4%) | 13 (92.9%) | 14 (100%) | 13 (92.9%) | 14 (100%) | 14 (100%) |  |
| Blood pressure instrument | 9 (64.3%) | 8 (57.1%) | 14 (100%) | 11 (78.6%) | 13 (92.9%) | 13 (92.9%) |  |
| Antibiotics mother | 8 (57.1%) | 13 (92.9%) | 14 (100%) | 13 (92.9%) | 13 (92.9%) | 14 (100%) |  |
| Thermometer | 8 (57.1%) | 13 (92.9%) | 14 (100%) | 7 (50%) | 13 (92.9%) | 12 (85.7%) |  |
| Vitamin K | 1 (7.1%) | 0 (0%) | 10 (71.4%) | 3 (21.4%) | 9 (64.3%) | 7 (50%) | Low availability (<50%) to mid-range availability (50-79%) |
| Antibiotics baby | 3 (21.4%) | 8 (57.1%) | 10 (71.4%) | 9 (64.3%) | 9 (64.3%) | 11 (78.6%) |  |
| Oxytocin | 5 (35.7%) | 6 (42.9%) | 9 (64.3%) | 9 (64.3%) | 9 (64.3%) | 8 (57.1%) |  |
| Magnesium Sulfate | 6 (42.9%) | 2 (14.3%) | 8 (57.1%) | 5 (35.7%) | 7 (50%) | 6 (42.9%) |  |
| HIV testing kit | 5 (35.7%) | 7 (50%) | 10 (71.4%) | 9 (64.3%) | 10 (71.4%) | 8 (57.1%) |  |
| Clean towel | 6 (42.9%) | 5 (35.7%) | 9 (64.3%) | 5 (35.7%) | 11 (78.6%) | 6 (42.9%) |  |
| Partograph | 1 (7.1%) | 1 (7.1%) | 2 (14.3%) | 0 (0%) | 4 (28.6%) | 2 (14.3%) | Low (<50%) availability that remains low |
| Nevirapine baby | 0 (0%) | 0 (0%) | 2 (14.3%) | 1 (7.1%) | 0 (0%) | 0 (0%) |  |
| Nevirapine mother | 0 (0%) | 0 (0%) | 2 (14.3%) | 1 (7.1%) | 0 (0%) | 1 (7.1%) |  |
| Fetoscope or doppler | 2 (14.3%) | 2 (14.3%) | 6 (42.9%) | 3 (21.4%) | 10 (71.4%) | 5 (35.7%) |  |

**^**Hand hygiene supplies refers to water and soap OR alcohol rub

**For example, on average at baseline 75% of intervention sites and 71% control sites had a cord tie - clamp available. At 6 months, 93.8% of intervention sites and 78.6% of control sites had a cord tie – clamp available.

Note: At 12 months, one low performing intervention site had closed after completing their survey at 6 months, resulting in exclusion of this site from analysis at 12 months, hence N=15; it’s matched pair was not within the bottom quartile of control sites.

**APPENDIX 3:** Source of available checklist supplies (N=23)* at intervention sites over time

8 & 9

*See Table 1 for list of the 23 items which were assessed for source of procurement if found during coach survey.

^Percent procured by patient: 4% (month 1), 5% (2), 4% (3), 5% (4), 5% (5), 5% (6), 5% (7), 6% (8-9).

Note: Three sites had one survey conducted in the ninth month so the eighth and ninth months of surveys were combined as the final month of measurement.

**APPENDIX 4:** Facility Supply Availability Survey (5 sections) conducted at both intervention and control sites

**
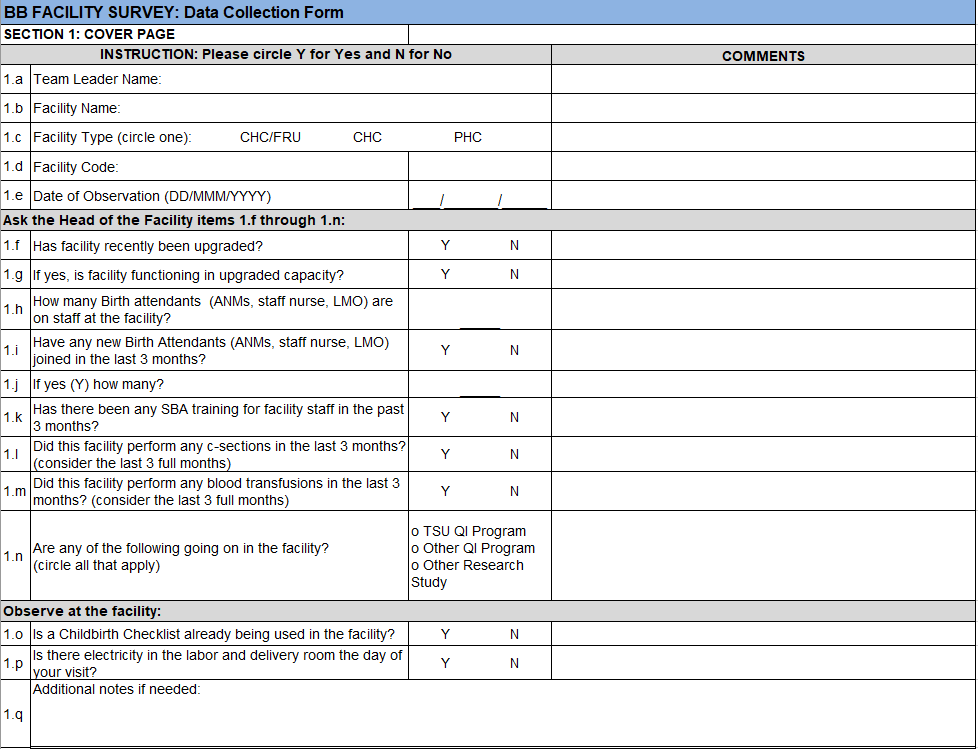
**

**
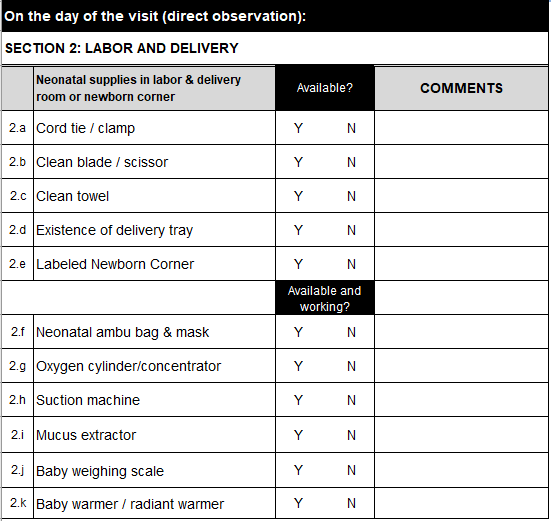
**

**
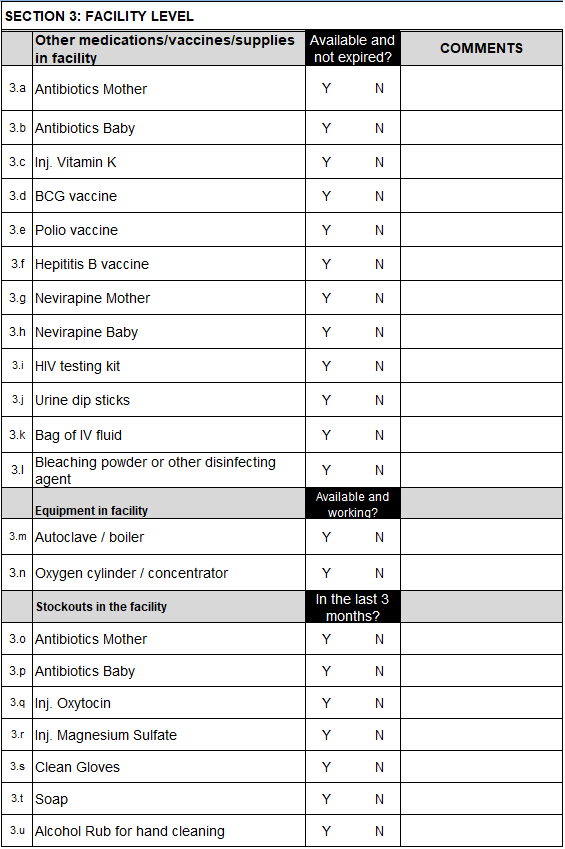
**

**
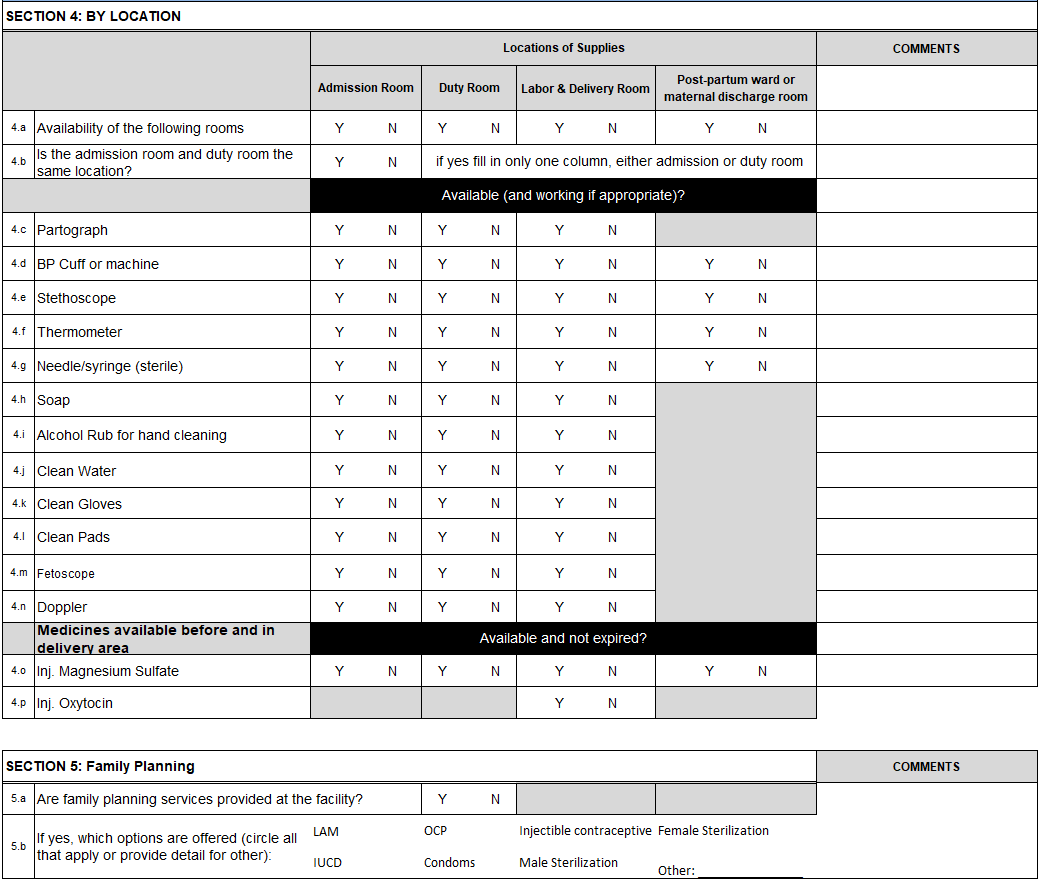
**

**APPENDIX 5:** Coach Supply Source Survey conducted at only intervention sites


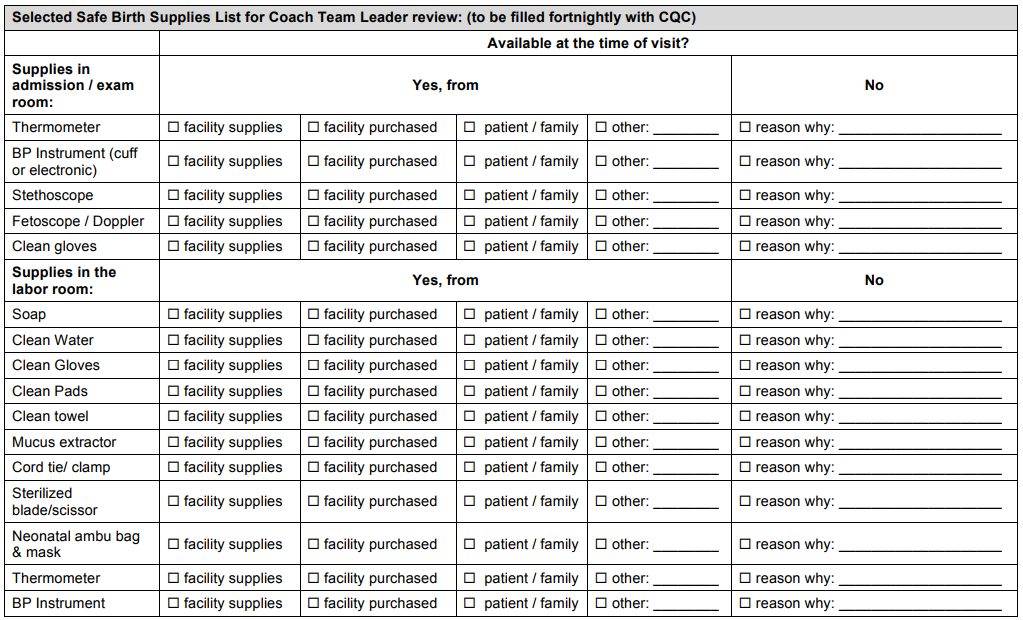


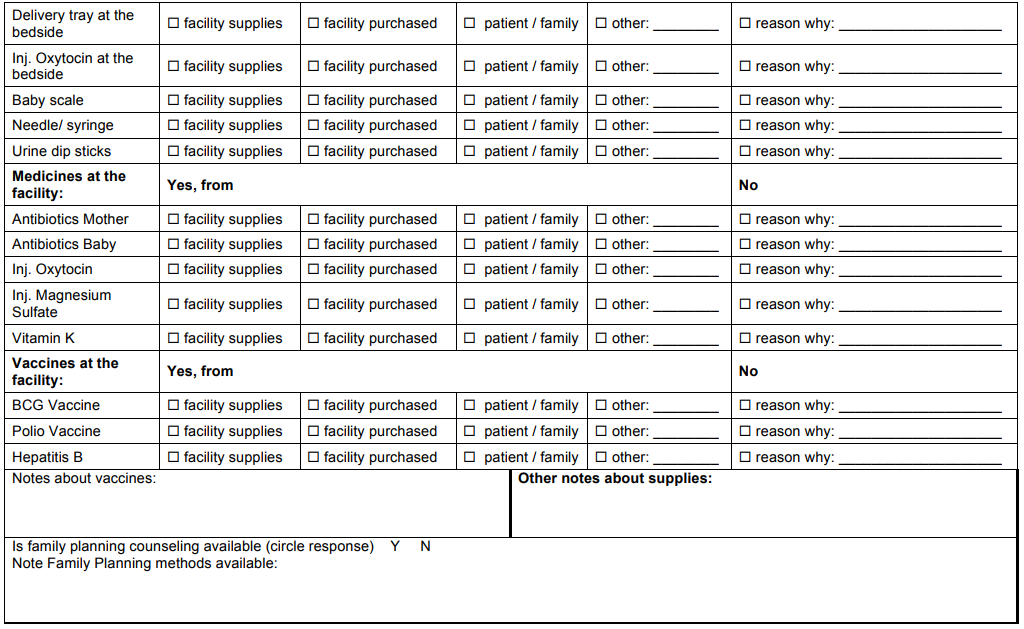


**APPENDIX 6:** Example of a coach supply source survey heat map used for quality improvement purposes during coaching at intervention sites

**
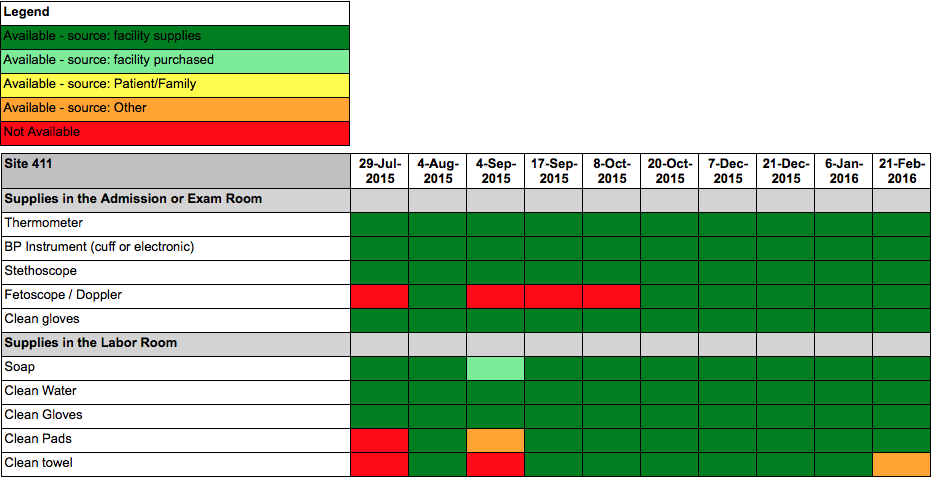
**
